# Supplementary material for: Role and mechanism of NCAPD3 in promoting malignant behaviors in gastric cancer
Source: Front Pharmacol. 2024 Apr 22;15:1341039. doi: 10.3389/fphar.2024.1341039 (PMC11070777; doi:10.3389/fphar.2024.1341039)
Supplement: Supplementary file 11 [file DataSheet2.ZIP › GSEA/Oncogenic signature/my_analysis.Gsea.1599461962066/ERBB2_UP.V1_DN.html]

Details for gene set ERBB2\_UP.V1\_DN[GSEA]

|  || Dataset | filtered\_dataset.sample\_info.cls#WT\_versus\_NCAPD3\_MUT |
| Phenotype | sample\_info.cls#WT\_versus\_NCAPD3\_MUT |
| Upregulated in class | WT |
| GeneSet | ERBB2\_UP.V1\_DN |
| Enrichment Score (ES) | 0.13243048 |
| Normalized Enrichment Score (NES) | 0.60874104 |
| Nominal p-value | 0.93658537 |
| FDR q-value | 0.99320954 |
| FWER p-Value | 1.0 |
Table: GSEA Results Summary

  

Fig 1: Enrichment plot: ERBB2\_UP.V1\_DN      
 Profile of the Running ES Score & Positions of GeneSet Members on the Rank Ordered List

  

| SYMBOL | TITLE | RANK IN GENE LIST | RANK METRIC SCORE | RUNNING ES | CORE ENRICHMENT || 1 | 23253 | ANKRD12 | 34 | 0.977 | 0.0527 | Yes |
| 2 | 3667 | IRS1 | 83 | 0.821 | 0.0829 | Yes |
| 3 | 55006 | TRMT61B | 101 | 0.782 | 0.1324 | Yes |
| 4 | 51018 | RRP15 | 192 | 0.685 | 0.1216 | No |
| 5 | 23255 | MTCL1 | 505 | 0.471 | -0.0663 | No |
| 6 | 23586 | DDX58 | 584 | 0.433 | -0.0883 | No |
| 7 | 9653 | HS2ST1 | 591 | 0.429 | -0.0588 | No |
| 8 | 7290 | HIRA | 612 | 0.417 | -0.0402 | No |
| 9 | 26953 | RANBP6 | 630 | 0.406 | -0.0204 | No |
| 10 | 178 | AGL | 711 | 0.368 | -0.0490 | No |
| 11 | 55003 | PAK1IP1 | 734 | 0.359 | -0.0366 | No |
| 12 | 3434 | IFIT1 | 743 | 0.354 | -0.0143 | No |
| 13 | 839 | CASP6 | 792 | 0.330 | -0.0229 | No |
| 14 | 4071 | TM4SF1 | 851 | -0.251 | -0.0449 | No |
| 15 | 5723 | PSPH | 859 | -0.267 | -0.0288 | No |
| 16 | 2317 | FLNB | 936 | -0.360 | -0.0552 | No |
| 17 | 3665 | IRF7 | 956 | -0.375 | -0.0393 | No |
| 18 | 9926 | LPGAT1 | 1036 | -0.434 | -0.0620 | No |
| 19 | 253959 | RALGAPA1 | 1066 | -0.456 | -0.0469 | No |
| 20 | 857 | CAV1 | 1098 | -0.475 | -0.0317 | No |
| 21 | 6947 | TCN1 | 1135 | -0.503 | -0.0179 | No |
| 22 | 7074 | TIAM1 | 1143 | -0.508 | 0.0172 | No |
| 23 | 9411 | ARHGAP29 | 1174 | -0.539 | 0.0381 | No |
| 24 | 10150 | MBNL2 | 1217 | -0.591 | 0.0545 | No |
| 25 | 6782 | HSPA13 | 1394 | -1.064 | 0.0115 | No |
Table: GSEA details [plain text format]

  

Fig 2: ERBB2\_UP.V1\_DN      
 Blue-Pink O' Gram in the Space of the Analyzed GeneSet

  

Fig 3: ERBB2\_UP.V1\_DN: Random ES distribution      
 Gene set null distribution of ES for **ERBB2\_UP.V1\_DN**
